# Ceramic-Based Dielectric Materials for Energy Storage Capacitor Applications

**DOI:** 10.3390/ma17102277

**Published:** 2024-05-11

**Authors:** Srinivas Pattipaka, Yeseul Lim, Yong Hoon Son, Young Min Bae, Mahesh Peddigari, Geon-Tae Hwang

**Affiliations:** 1 Department of Materials Science and Engineering, Pukyong National University, 45, Yongso-ro, Nam-Gu, Busan 48513, Republic of Korea; cnuphy444@gmail.com (S.P.); yesle912@naver.com (Y.L.); mike4009@pukyong.ac.kr (Y.H.S.); dud1560@pukyong.ac.kr (Y.M.B.); 2 Department of Physics, Indian Institute of Technology Hyderabad, Kandi 502284, Telangana, India; mahesh.p@phy.iith.ac.in

**Keywords:** ceramic-based dielectric materials, polarization, breakdown strength, recoverable energy density, energy efficiency, energy storage capacitors

## Abstract

Materials offering high energy density are currently desired to meet the increasing demand for energy storage applications, such as pulsed power devices, electric vehicles, high-frequency inverters, and so on. Particularly, ceramic-based dielectric materials have received significant attention for energy storage capacitor applications due to their outstanding properties of high power density, fast charge–discharge capabilities, and excellent temperature stability relative to batteries, electrochemical capacitors, and dielectric polymers. In this paper, we present fundamental concepts for energy storage in dielectrics, key parameters, and influence factors to enhance the energy storage performance, and we also summarize the recent progress of dielectrics, such as bulk ceramics (linear dielectrics, ferroelectrics, relaxor ferroelectrics, and anti-ferroelectrics), ceramic films, and multilayer ceramic capacitors. In addition, various strategies, such as chemical modification, grain refinement/microstructure, defect engineering, phase, local structure, domain evolution, layer thickness, stability, and electrical homogeneity, are focused on the structure–property relationship on the multiscale, which has been thoroughly addressed. Moreover, this review addresses the challenges and opportunities for future dielectric materials in energy storage capacitor applications. Overall, this review provides readers with a deeper understanding of the chemical composition, physical properties, and energy storage performance in this field of energy storage ceramic materials.

## 1. Introduction

**Figure 1:**
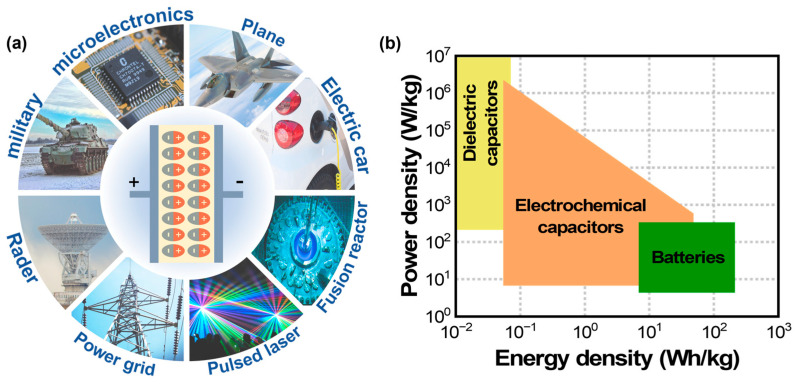
(**a**) Various applications of dielectric capacitors in power electronics and pulse power applications. (**b**) Comparison of the power density versus energy density of batteries, electrochemical capacitors, and dielectric capacitors.

Energy storage devices such as batteries, electrochemical capacitors, and dielectric capacitors play an important role in sustainable renewable technologies for energy conversion and storage applications [1,2,3]. Particularly, dielectric capacitors have a high power density (~10^7^ W/kg) and ultra-fast charge–discharge rates (~milliseconds) when compared to electrochemical capacitors and batteries (Figure 1b) [2,3,4,5,6,7,8,9,10,11,12,13]. These advantages of dielectric capacitors make them promising for applications in power electronics and pulsed power systems, as shown in Figure 1a. For instance, more than three trillion multilayer ceramic capacitors (MLCCs) are manufactured annually and are used in cell phones or electric vehicles [6,7,8,9,14,15]. However, dielectric capacitors have a lower energy storage density of 10^−2^ to 10^−1^ Wh/kg than electrochemical capacitors and batteries, which limits their practical applications. Therefore, high-performance dielectric materials in terms of high energy storage density, high energy efficiency, fast charge–discharge capabilities, better thermal or frequency stability, fatigue resistance, lifetime reliability, equivalent series resistance, and low manufacturing costs are needed for power electronics and pulse power applications.

The storage performance depends on the charge accumulation in dielectric materials, which are a key component of capacitors. Dielectric materials, including organic (polyvinylidene fluoride (PVDF), biaxially oriented polypropylene (BOPP), polyimide (PI), etc.), and inorganic (ceramics, glass, and glass-based ceramics) materials, have been widely investigated to improve the energy storage performance [9,16,17,18,19,20]. In recent years, significant improvements to dielectric materials have been made, although each material still has limitations. The polymers offer a high breakdown strength (BDS), low relative dielectric permittivity, and weak thermal stability, making dielectric materials for energy storage a long-term goal. Meanwhile, ceramic-based dielectric materials are popular research topics due to their application in energy storage, adaptability to various environments, fundamentality, and other factors. Therefore, the topic of dielectrics will be discussed further in this review. These materials are classified into linear dielectrics (LDs), ferroelectrics (FEs), antiferroelectrics (AFEs), and relaxor ferroelectrics (RFEs) [17,20]. They are considered viable candidates for energy storage due to their differing properties in BDS and polarization, which primarily influence energy storage performance.

This review paper presents fundamental concepts of energy storage in dielectric capacitors, including an introduction to dielectrics and key parameters to enhance energy storage responses. We also summarize recent progress in dielectrics, such as bulk ceramics, ceramic films, and multilayer ceramic capacitors, including the phase, local structure, microstructure, domain evolution, layer thickness, stability, and electrical homogeneity; fabrication methods, dopants/composites, and various strategies for enhancing energy storage properties in dielectric capacitors are also briefly discussed.

## 2. Fundamental Concepts for Energy Storage in a Dielectric Capacitor

### 2.1. Dielectric Capacitor

**Figure 2:**
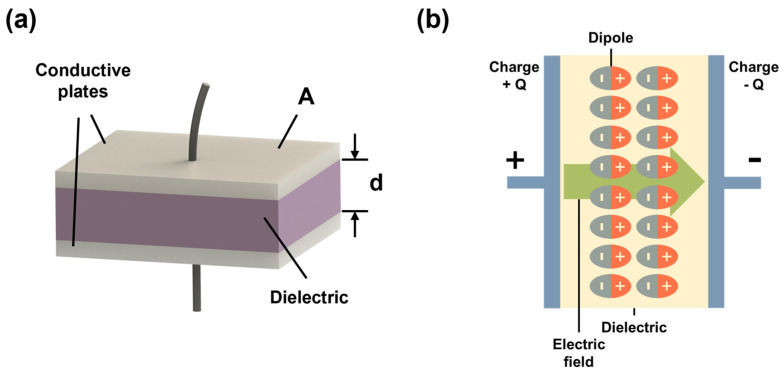
Schematic diagram of (**a**) a dielectric capacitor, and (**b**) a dielectric between two conductive plates, where electric dipoles are displaced and oriented by the applied electric field due to polarization.

A parallel plate capacitor is composed of two parallel conducting plates that are separated by a ceramic layer, as schematically shown in Figure 2. When a dielectric capacitor is placed in an external electric field, the electric dipoles will be displaced and oriented due to polarization (Figure 2b). The capacitance of a dielectric capacitor (*C*) is the ability to store electric charge and is given by the following equation:

(1) $$C=\frac{Q}{V}$$

where *Q* is the charge and *V* is the voltage applied to the capacitor.

According to Gauss’s law,

(2) $$V=\frac{Qd}{\varepsilon_{0}\varepsilon_{r}A}$$

The capacitance of a parallel plate capacitor can be calculated in terms of the sample area and thickness via comparing Equations (1) and (2).

(3) $$C=\varepsilon_{0}\varepsilon_{r}\frac{A}{d}$$

where *ε*_0_ is the permittivity of free space, *ε_r_* is relative dielectric permittivity, *A* is the area of metal plates, and *d* is the thickness of the ceramic sample (Figure 2a).

### 2.2. Evaluation of Energy Storage Performance

**Figure 3:**
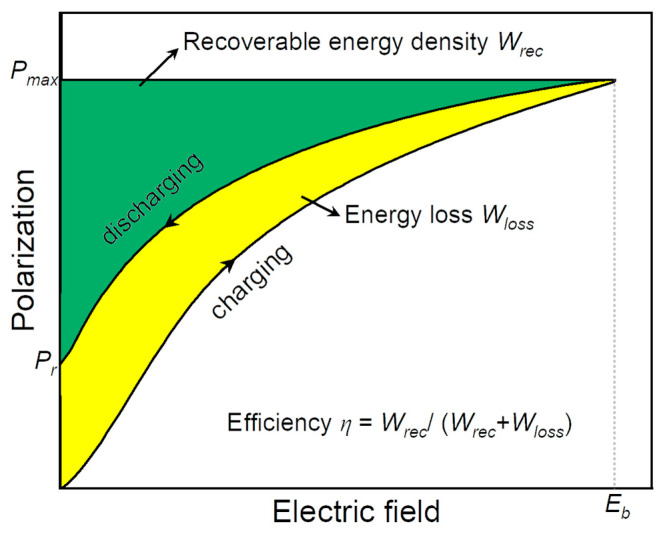
Schematic of the recoverable energy density and energy loss from the *P-E* hysteresis loop of a ceramic capacitor.

The energy storage density (*W*) of a linear dielectric material is determined with the following equation [21]:

(4) $$W=\frac{1}{2}\varepsilon_{0}\varepsilon_{r}E^{2}$$

where *ε*_0_ is the permittivity of free space, *ε_r_* is dielectric permittivity, and *E* is the applied electric field. In contrast, the nonlinear dielectric materials (FEs, AFEs, and RFEs) exhibit energy loss. Therefore, the total energy storage density (*W_tot_*), recoverable energy density (*W_rec_*), and energy storage efficiency (*η*) of these materials are calculated from the hysteresis loops as follows [22,23,24]:

(5) $$W_{tot}=\int_{0}^{P_{m}}EdP\;(Charging)$$

(6) $$W_{rec}=\int_{P_{r}}^{P_{m}}EdP\;(Discharging)$$

(7) $$\eta =\frac{W_{rec}}{W_{rec}+W_{loss}}\times 100\%$$

where *E* is the applied electric field, *P* is polarization, *P_max_* is maximum polarization, *P_r_* is remnant polarization, and *W_loss_* is energy loss, as schematically shown in Figure 3.

### 2.3. Key Parameters for Energy Storage Performance

#### 2.3.1. Energy Storage Density and Efficiency

*W_rec_* and *η* are the most important parameters for evaluating the energy storage performance of dielectric materials, which are related to dielectric permittivity and polarization. A high *W_rec_* of dielectric materials means that more energy can be stored in a given volume, promoting miniaturization and lightweight and low-cost materials being utilized in consumer power electronics and pulse power systems. It can be concluded from Equations (4)–(7) and Figure 3 that a higher *ε_r_*, *P_max,_* and BDS lead to higher energy density, whereas low dielectric or hysteresis losses and low *P_r_* improve energy storage efficiency in dielectric materials. Moreover, the material should have low electronic or ionic conductivity to resist higher electric fields.

#### 2.3.2. Polarization Difference

The energy storage density and efficiency of a ceramic capacitor’s are mostly related to the shape of the *P-E* loop due to the area under the curve providing the *W_rec_* (Figure 3). Therefore, the energy storage performance depends on the value of Δ*P* (Δ*P = P_max_*− *P_r_*), and the *W_rec_* increases with Δ*P* [25,26]. However, some of the stored energy in dielectrics will be dissipated during the depolarization/discharge process, which will be equal to the area of the *P-E* loop (i.e., *W_loss_* can be seen in Figure 3) [27,28]. Such energy loss causes heat generation, consequently deteriorating the capacitor’s thermal stability and lifespan. The heat generation is attributed to the dielectric loss (*tan*δ) and temperature rise (ΔT), as provided using the following equations:

(8) $$w_{h}=\pi \varepsilon_{0}\varepsilon_{r}E^{2}tan\delta$$

(9) $$∆T=\frac{fV_{e}}{hA}W_{h}$$

where *f* is the driving frequency, *V_e_* is the effective volume to an applied electric field, *h* is the heat transfer coefficient, and *A* is the total surface area of a sample [27,28]. Therefore, a low *tan*δ and a large Δ*P* (i.e., low *P_r_* and high *P_max_*) are critical parameters for achieving high energy storage performance in ceramic capacitors.

#### 2.3.3. Dielectric Breakdown Strength

The energy storage response of ceramic capacitors is also influenced by the *E_b_*, as the *W_rec_* is proportional to the *E*, as can be seen in Equation (6) [29]. The BDS is defined as the maximum electric field over which the electrical resistance of a dielectric significantly decreases. The *E_b_* of these capacitors strongly depends on intrinsic (bandgap, grain size, phase, defect dipoles, material thickness, microstructure, and porosity) and extrinsic (working/environmental conditions and electrode configuration) properties [5,30,31,32,33]. Therefore, a dense microstructure is a critical factor for the fabrication of a high-quality ceramic capacitor to achieve greater capacitance under high electric fields. However, dielectric breakdown is caused by pores, cracks, interfaces, and compositional inhomogeneity [31,33,34]. The porosity in dielectrics affects the dielectric breakdown strength and can cause overheating and thermal stress, resulting in breakdowns at higher electric fields [31,33].

#### 2.3.4. Discharge Time

The discharge time is another critical parameter for energy storage. The discharging speed of a ceramic capacitor is calculated in terms of the discharge time, represented by *τ*_0.90_. It is defined as the time required for a capacitor to discharge 90% of its stored energy. The discharge time is 0.15 µs at an infinite time, and it depends on the dielectric permittivity and thickness of the material, load resistance, and applied voltage [6]. The discharge time should be very short for pulsed power energy storage capacitor applications.

#### 2.3.5. Reliability

Dielectric capacitors are interconnected with their embedded system and operating conditions, influenced by various factors such as temperature, frequency, and voltage fluctuations. Therefore, better reliability, often called high electric fatigue endurance, protects the physical integrity of pulsed power systems, particularly dielectric capacitors, during energy storage under harsh circumstances. The evaluation of the resistance to stimuli can be conducted through observing the distinct features of *P-E* loops under particular investigation conditions.

### 2.4. Categories of Dielectric Materials

**Figure 4:**
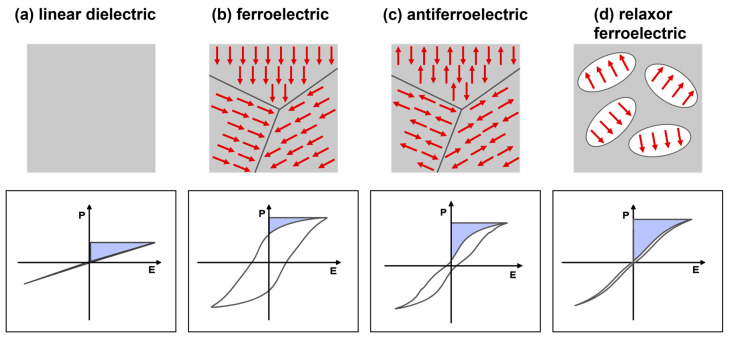
Schematic of the electric field-dependent polarization response and ferroelectric domain structures with dipole orientation for (**a**) LDs, (**b**) FEs, (**c**) AFEs, and (**d**) RFEs.

Based on polarization versus the electric field response, dielectric materials are categorized into linear dielectrics (LDs) and nonlinear dielectrics (NLDs), such as ferroelectrics (FEs), anti-ferroelectrics (AFEs), and relaxor ferroelectrics (RFEs). Figure 4a–d show a schematic of the electric field-dependent polarization response and corresponding ferroelectric domain structures with dipole orientation for the LDs and NLDs. LDs display an almost linear polarization response via the application of an electric field, owing to the lack of permanent dipoles (Figure 4a). Ferroelectric materials display superior polarization responses even in the absence of an external electric field due to the presence of a net dipole moment. Therefore, they have a strong nonlinear relation with the applied electric field (Figure 4b). In AFE materials, the adjacent dipoles are oriented in antiparallel directions, resulting in zero net polarization. They show double hysteresis loops at higher electric fields caused by AFE to FE phase transitions (Figure 4c). In RFEs, the existence of polar nanoregions (PNRs)/nanodomains greatly reduces cooperative coupling between ferroelectric domains, which limits spontaneous polarization and, consequently, slim *P-E* loops (Figure 4d).

### 2.5. Energy-Storage Mechanism of the Materials

Ferroelectric materials are a fascinating class of dielectrics with unique properties, making them promising in the field of energy storage, conversion, and harvesting applications due to their electrical, mechanical, and thermal properties being intrinsically interrelated. All ferroelectrics are piezoelectric and pyroelectric materials, which make ferroelectrics extremely useful in multiple applications. The coupling of ferroelectric polarization to temperature, stress, and electric field enables various energy storage and conversion approaches that rely on diverse stimuli. The polarization is used in three ways, namely capacitive-energy storage (i.e., energy is stored in the form of polarization), piezoelectric-energy harvesting (i.e., vibration-induced stress on a piezoelectric material is converted into charge via a change in polarization), and pyroelectric-energy conversion (i.e., thermodynamic cycles can be utilized to convert temperature fluctuations into current) [35].

Based on the energy storage mechanism and the charge–discharge process, there is a substantial variation in the power density and energy density in dielectric capacitors, electrochemical capacitors, and batteries (see Figure 1b). Batteries offer higher energy density, but lower power density because of the slow movement of charges, which are used for long-term, stable energy supplies and applications with a maximum of 5 V [2,3,12,36]. Electrochemical capacitors have moderate power density and energy storage density with a slow charge–discharge rate and a low operating voltage (<3 V) [36]. Dielectric capacitors have high power density but limited energy storage density, with a more rapid energy transfer than electrochemical capacitors and batteries; this is because they store energy via dielectric polarization in response to the external electrical fields rather than chemical reactions [3,12,13,35]. Therefore, dielectric capacitors have received great interest due to their low price and high operating voltages (kV/MV range) for longer durations, making them ideal for a wide range of applications, including consumer electronics and advanced pulsed power devices.

## 3. Dielectric Materials for Energy Storage

### 3.1. Bulk Ceramics

#### 3.1.1. Linear Dielectrics

LDs exhibit low energy loss, low relative dielectric permittivity, and a high breakdown electric field, and are promising for energy storage device applications under certain working conditions. Various LDs, such as Al_2_O_3_ [37], TiO_2_ [38], SrTiO_3_ (ST) [39,40,41], and CaTiO_3_ (CT) [41,42,43,44,45], have been reported to improve their energy storage performances. Pure ST ceramics exhibited a relative dielectric permittivity of 300, a breakdown electric field of 1600 kV/mm, and a dielectric loss of 0.01 at RT, and are utilized for integrated circuit applications [39,42,46]. Chemical modifications have been adopted to enhance the energy storage properties in ST ceramic capacitors. Notably, 2 mol% of Ca doping in the ST system was improved energy density of 1.95 J/cm^3^ and an efficiency of 72.3% at a breakdown field of 333 kV/cm, which is nearly three times higher than pure ST [41]. These improved energy storage properties in titanium-based ceramics are attributed to the insulation attenuation property caused by electronic hopping from the valence band to the conduction band. The substitution of Zr ions at the Ti site of Sr_0.98_Ca_0.02_TiO_3_ boosted the energy storage density to 2.77 J/cm^3^ and yielded an efficiency of 77.7% by reducing the dielectric loss and leakage current density, which is attributable to the higher chemical durability [47]. Mg-doped ST ceramics showed an enhanced *W_rec_* of 1.86 J/cm^3^ and *η* of 72.3% at a BDS of 362 kV/cm by lowering the dielectric loss to 0.001 with a moderate dielectric constant of 280 [45]. Interestingly, a binary composite of CaZrO_3_-0.05SrTiO_3_ exhibited a high *W_rec_* of 5 J/cm^3^ at 1000 kV/cm, caused by a low dielectric loss of 0.001 and dielectric constant of 35 [48]. It is well known that the BDS is directly proportional to the bandgap energy, and a higher bandgap energy enables a higher BDS [43,48]. Shay et al. [43] reported a binary composition of 0.8CaTiO_3_-0.2CaHfO_3_ (with 0.5 mol% of Mn doping) by modulating their bandgap energies, and showed a high *W_rec_* of 9 J/cm^3^ at 1200 kV/cm (9.6 J/cm^3^ at 1300 kV/cm). In a similar vein, BaZrO3-CaTiO_3_ and SrZrO_3_-CaTiO_3_ binary compositions have shown improved energy storage performance [43,48].

#### 3.1.2. Ferroelectrics

**Figure 5:**
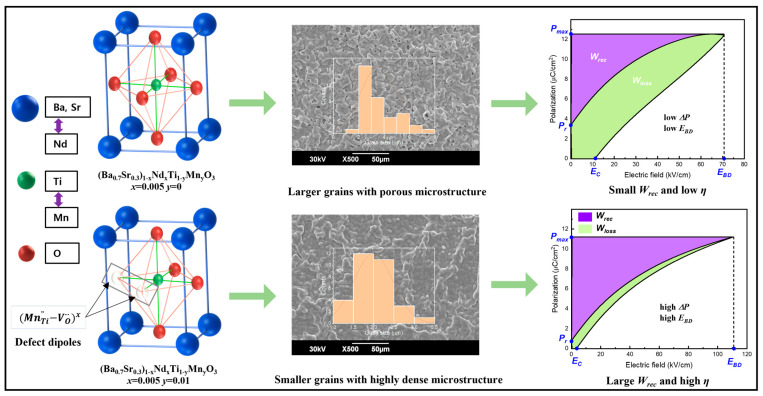
Schematic illustration of a defect dipole concept to achieve energy storage properties of Nd and Mn-co-doped Ba_0.7_Sr_0.3_TiO_3_ ceramics. Defect dipoles between donor/acceptor ions and oxygen vacancies capture electrons, decrease grain size, and enable a high difference between *P_max_* and *P_r_*, thereby enhancing the BDS with Nd and Mn, which results in an improved *W_rec_* and *η* in Ba_0.7_Sr_0.3_TiO_3_ ceramics. Reproduced with permission [50]. Copyright 2023, MDPI.

In comparison with LDs, FE materials show strong nonlinear behavior with high polarization, high dielectric permittivity, high energy loss, and a low BDS. Various rare earth elements and dopants (such as Sr, Ca, Nd, Mn, and Zr) were substituted at A/B-sites of the BT system to enhance the BDS and energy storage responses. Sr-doped BT (Ba_1−*x*_Sr*_x_*TiO_3_, BST) ceramics were investigated, showing a high dielectric constant of 650, a low dielectric loss of 7.6 × 10^−4^ @ 1kHz, a low *W_rec_* of 0.23 J/cm^3^, and the Curie temperature being lowered far below RT [49]. Choi et al. [50] reported a defect dipole engineering method to enhance the energy storage performance by co-doping Nd and Mn in Ba_0.7_Sr_0.3_TiO_3_ ceramics. Figure 5 presents a schematic illustration of a defect dipole concept between acceptor ions and oxygen vacancies in Ba_0.7_Sr_0.3_TiO_3_ ceramics. These defect dipoles with a uniform and small-grained microstructure enable a high difference between *P_max_* and *P_r_* (Δ*P*~10.39 µC/cm^2^) and capture electrons, improving the BDS to 110.6 kV/cm with co-doping of Nd and Mn; this in turn leads to improvements in the *W_rec_* to 0.41 J/cm^3^ and a high *η* of 84.6% in Ba_0.7_Sr_0.3_TiO_3_ ceramics. Interestingly, Dong et al. [33] reported 1.6 wt% ZnO doped in Ba_0.3_Sr_0.7_TiO_3_ ceramics with an enhanced *W_rec_* of 3.9 J/cm^3^ at 40 kV/mm. Taking a theoretical approach, Wang et al. [51] reported first-principles calculations and molecular dynamic simulations to study the effects of the chemical composition, phase under temperature, and electric fields on the ferroelectric and energy storage properties of ABO_3_ perovskite FEs. These simulation results revealed a *W_rec_* of 2.8 J/cm^3^ and a *η* of 95% at *E_b_* of 350 kV/cm in Ba_0.6_Sr_0.4_TiO_3_ ceramics, and, furthermore, a *W_rec_* of 30 J/cm^3^ and a *η* of 92% obtained at an *E_b_* of 2750 kV/cm in the same composition of Ba_0.6_Sr_0.4_TiO_3_. However, practically, a BDS on the order of a thousand kV/cm is not achievable in most FEs because of numerous defects, an internal mechanical field, internal stress, and the influence of crystallographic lattice constants, phase transition, and grain size. Song et al. [52] reported the effect of grain sizes from 0.5 µm to 5.6 µm in Ba_0.6_Sr_0.4_TiO_3_ ceramics to investigate the energy storage performance, and the samples with a grain size of 0.5 µm showed a high *W_rec_* of 1.28 J/cm^3^ at an *E_b_* of 243 kV/cm.

#### 3.1.3. Anti-Ferroelectrics

Antiferroelectric materials differ from typical ferroelectrics in their distinctive crystal structure, with adjacent diploes aligned in opposite orientations. To generate a strong ferroelectric state, diploes are subjected to a high electric field in order to realign their polarization orientation. This results in the formation of double hysteresis loops which consist of a linear polarization response in the AFE state and a ferroelectric hysteresis loop in the FE state. The huge reversible polarization would increase the energy storage density. However, thermal runaway and high energy dissipation due to hysteresis remain major challenges in building high energy density AFEs. To improve energy storage properties, enhancing the linear polarization response area and decreasing hysteresis loss by changing the phase transition parameters is recommended.

**Figure 6:**
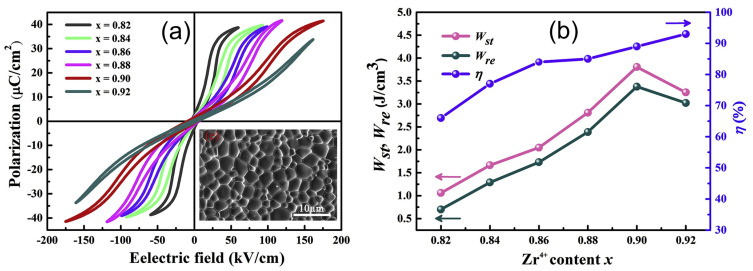
(**a**) P-E loops and (**b**) *W_st_*, *W_re_*, and *η* of the (Pb_1−y_La_y_)(Zr*_x_*Ti_1−*x*_)O_3_ ceramics for *y* = 0.07 and *x* = 0.82 to 0.92. (**c**) shows a SEM image for *x* = 0.9. Reproduced with permission [55]. Copyright 2019, Elsevier.

PbZrO_3_ (PZ) AFE materials have been widely investigated due to their diverse phase transition features [53]. Chemical substitution affects reform polarization properties by altering the switching electric field between the AFE and FE phases. As per the phase diagram of La_2_O_3_-PbZrO_3_-PbTiO_3_ [54], Peixin et al. [55] reported the energy storage properties with the substitution of Ti^4+^ with Zr^4+^ at the B-site of in (Pb_1−y_La_y_)(Zr*_x_*Ti_1−*x*_)O_3_ (PLZT) ceramics. The substitution of Zr^4+^ at Ti^4+^ can decrease the tolerance factor and improve the AFE properties. The *P-E* loops of PLZT AFEs become very slim with the substitution of the Zr concentration, and a high *W_rec_* of 3.38 J/cm^3^ and a high *η* of 86.5% were achieved with the optimized composition of *x* = 0.9 and *y* = 0.07 (Figure 6). Similarly, the substitution of La^3+^ at Pb^2+^ (the A-site) of (Pb_1−1.5*x*_La*_x_*)(Zr_0.5_Sn_0.43_Ti_0.07_)O_3_ improved the AFE phase stability and provided slim P-E loops, resulting in the highest *W_rec_* of 4.2 J/cm^3^ and a high *η* of 78% for the *x* = 0.03 composition [56]. On the basis of the phase diagram of PbZrO_3_-PbTiO_3_-PbSnO_3_ [57], Wang et al. [58] reported field-induced multiphase transitions (AFE-FE and FE-FE) at weak and high electric fields in (Pb_0.98_La_0.02_)(Zr_0.55_Sn_0.45_)_0.995_O_3_ AFE ceramics, yielding superior energy storage properties of a *W_rec_* of 10.4 J/cm^3^ and a *η* of 87% at 400 kV/cm. Moreover, Liu et al. reported the substitution of Sr^2+^ in (Pb_0.98-*x*_La_0.02_Sr*_x_*)(Zr_0.9_Sn_0.1_)_0.995_O_3_ AFE ceramics to improve the BDS and the switching of electric fields between the AFE and FE phase, resulting in an ultrahigh *W_rec_* of 11.18 J/cm^3^ and a high *η* of 82.2% [59].

**Figure 7:**
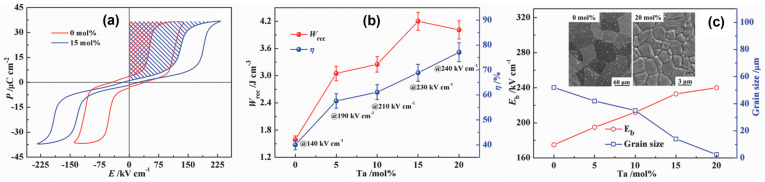
(**a**) *P-E* loops of Ag(Nb_1−*x*_Ta*_x_*)O_3_ ceramics for *x* = 0 and 0.15, (**b**) *W_re_* and *η*, and (**c**) *E_b_* and grain size of Ag(Nb_1−*x*_Ta*_x_*)O_3_ ceramics for *x* = 0 to 20. The inset of Figure 7c shows SEM images of Ag(Nb_1−*x*_Ta*_x_*)O_3_ ceramics for *x* = 0 and 0.20. Reproduced with permission [26]. Copyright 2017, Wiley-VCH.

In spite of the excellent features of AFE lead-based ceramics, various AFE lead-free ceramics have garnered attention due to environmental concerns. Zhao et al. [26] reported lead-free AFE AgNbO_3_ (AN) ceramics with a Ta substitution to improve their energy storage properties. Figure 7 presents the *P-E* loops of pure, Ta-doped AN ceramics and energy storage properties of Ag(Nb_1−*x*_Ta*_x_*)O_3_ ceramics as a function of the Ta concentration (*x* = 0 to 20 mol%). A high *W_rec_* of 4.2 J/cm^3^ (260% higher than that of pure AN) and a *η* of 69% were achieved in Ag(Nb_1−*x*_Ta*_x_*)O_3_ ceramics for *x* = 0.15. The substitution of Ta into the Nb site improves antiferroelectricity due to the lower polarizability of B-site cations, and also reduces grain size and enhances density, resulting in a high BDS of 240 kV/cm (Figure 7c). Researchers recently investigated the underlying mechanism between AFE properties and the energy barrier (EB), where increased and decreased EB for the AFE-FE phase transition via the doping of Sm^3+^, Ca^2+^, and the co-doping of Sm^3+^/Ta^5+^ at the A- and A/B-sites of AN-based ceramics, which exhibited high *W_recs_* of 5.2, 4.87, and 3.55 J/cm^3^, respectively [60,61,62]. Luo et al. [63] reported a high *W_rec_* of 6.3 J/cm^3^ and a high *η* of 90%, realized by the M_2_-M_3_ phase boundary, the stabilized AFE phase, the presence of relaxor properties, and slim double *P-E* loops. In a similar way, Li et al. [5] reported 0.55(Bi_0.5_Na_0.5_)TiO_3_-0.45(Bi_0.2_Sr_0.7_)TiO_3_ relaxor-antiferroelectric ceramics with a *W_rec_* of 2.5 J/cm^3^ for bulk ceramics and 9.5 J/cm^3^ for multilayer ceramic capacitors, respectively. In addition, Qi et al. [64,65] fabricated 0.78(Bi_0.5_Na_0.5_)TiO_3_-0.22NaNbO_3_ and 0.76NaNbO_3_–0.24(Bi_0.5_Na_0.5_)TiO_3_ relaxor-antiferroelectric ceramics with giant energy storage properties as follows: a *W_rec_* of 7.02 and 12.2 J/cm^3^ and a *η* of 85% and 69%, respectively. Instead of the chemical substitution/composition method, Wang et al. [66] utilized a hydrothermal method to enhance the energy storage performance of AN ceramics and form a fine-grain size of 3 µm, which resulted in a high BDS of 250 kV/cm.

#### 3.1.4. Relaxor Ferroelectrics

Relaxor ferroelectric materials, a significant subclass of ferroelectric materials, have drawn the attention of researchers because of their intriguing and little-known physics since Smolenskii’s first discovery of the relaxor properties in a BaTiO_3_ (BT)-based system [67]. The RFEs are thought to be the most promising energy storage materials for applications in electrostatic energy storage because of their distinct and slim P-E loops, in contrast with regular ferroelectrics, and are beneficial for energy storage. It has been established that the vast differences between RFEs and FEs are closely related to the dynamics of their domain structure. The nanodomains/PNRs, which range in size from several nm to µm and are more responsive to external electric fields, are predicted to facilitate a moderate *P* and slight *P_r_* in RFEs, and these features are expected to contribute to a high *W_rec_* and *η* [68]. In this regard, various lead-based and lead-free perovskite RFEs, namely (Pb(Zn_1/3_Nb_2/3_)O_3_-PbTiO_3_ (PZN-PT) [69,70], Pb(Mg_1/3_Nb_2/3_) O_3_-PbTiO_3_ (PMN-PT) [70], (Pb, La)(Zr, Ti)O_3_ (PLZT) [71] and BT [72,73,74], (Na, K)NbO_3_ (KNN) [75,76], and (Bi, Na)TiO_3_ (BNT) [75,76], have been explored for energy storage applications, respectively.

In lead-based RFEs, the PLZT has received strong attention for energy storage applications because of their phase structure (paraelectric phase, rhombohedral FEs, tetragonal FEs, orthorhombic AFEs, and RFEs) through chemical composition design. It is observed that relaxor properties showing slim P-E loops can be obtained via the formation of a pseudocubic structure with a c/a ratio approaching one when exceeding 7 mol% of La^3+^ ions [77]. Thick/thin films have been fabricated to improve the BDS of the PLZT system. Hao et al. fabricated PLZT bulk ceramics with a thickness of 1 mm using a sol–gel synthesis process and an enhanced *W_rec_* of 28.7 J/cm^3^ and a *η* of 60% with a La:Zr:Ti ratio of 9:65:35 [78]. Furthermore, a Mn-doped PLZT thick film with the same ratio and same thickness showed a high *W_rec_* of 30.8 J/cm^3^ and a *η* of 68.4% at an electric field of 1185 kV/cm [79,80]. To date, the energy storage properties of PLZT with other lead-based RFEs and various chemical compositions have been reported, such as PZN-PT, PMN-PT, and Pb(Sn,Ti)O_3_ (PST), exhibiting *W_rec_* values ranging from 1 to 50 J/cm^3^ for energy storage device applications [81,82,83,84,85]. However, the utilization of lead-based dielectrics has a strong impact on human health and the environment due to their toxicity. Thus, researchers have been developing lead-free RFEs for energy storage applications.

**Figure 8:**
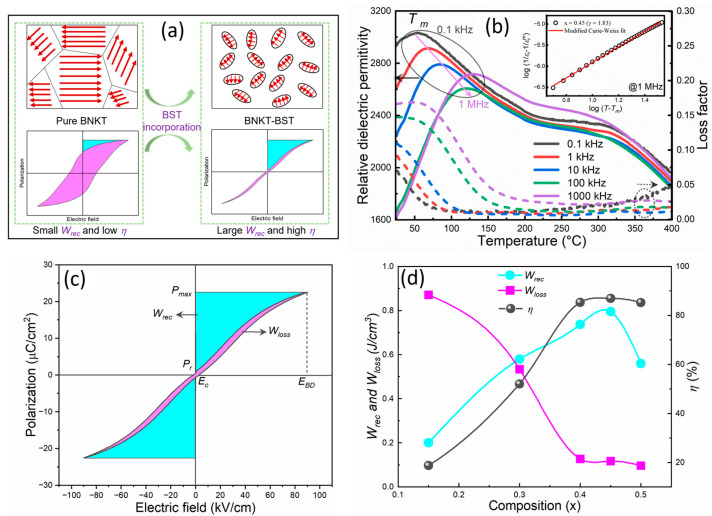
(**a**) Schematic of the domain structure and formation of the FE to RFE transition with the incorporation of BST into BNKT, leading to improved *W_rec_* and *η* (where the red arrows indicate the dipole orientation). (**b**) Temperature dependence of the relative dielectric permittivity and loss factor of 0.55BNKT-0.45BST composition. The inset of Figure 8b presents the $log\left(T-T_{m}\right)$ versus log1εr−1εrm of 0.55BNKT-0.45BST at 1 MHz. (**c**) *P-E* hysteresis loop of 0.55BNKT-0.45BST ceramics. (**d**) Composition versus *W_rec_*, *W_loss_*, and *η* for *x* = 0.15–0.50. Reproduced with permission [100]. Copyright 2023 MDPI.

**Figure 9:**
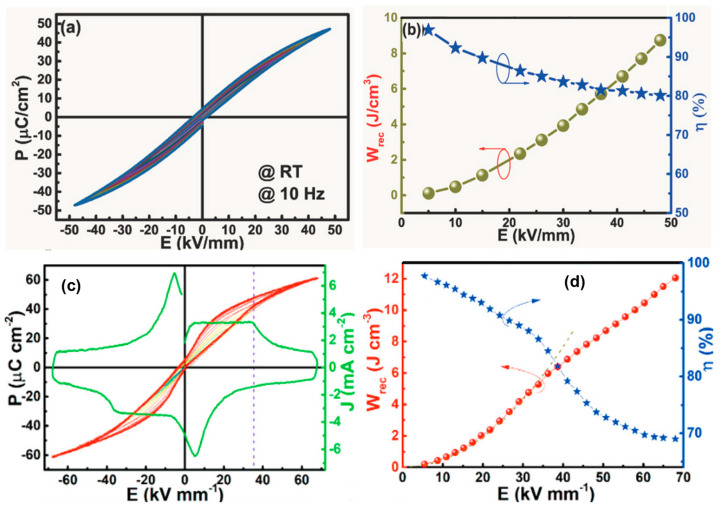
(**a**) *P-E* loops and (**b**) *W_rec_* and *η* values of 0.68 NN-0.32BLT ceramics at various fields. (**c**) P−E loops along with the current density versus electric field curve. Reproduced with permission [105]. Copyright 2021 John Wiley and Sons. (**d**) *W_rec_* and *η* values of 0.76 NN-0.24BNT ceramics at various fields and measured at 10 Hz and RT. Reproduced with permission [65]. Copyright 2019 John Wiley and Sons.

Over the past 20 years, since dielectric constant/polarization is independent of the applied electric field, temperature, and frequency, lead-free BT-based and weakly coupled RFEs have been explored in efforts to achieve high energy density and high efficiency based on the domain tailoring concept [4,23,86,87,88,89,90,91,92,93,94]. Ogihara et al. [86] reported a high *W_rec_* of 6.1 J/cm^3^ at 73 kV/mm in BT-BiScO_3_ thick films that were sustained until 300 °C. Yuan et al. [93] reported a domain evaluation using chemical composition and improvements in the energy storage of BT-based ceramics. Furthermore, lead-free BNT-based and strongly coupled RFEs with a high polarization response via minimizing hysteresis loss and leakage currents have been reported. Qiao et al. [95] demonstrated a high *W_rec_* of 4.14 J/cm^3^ in a Sr and La-co-doped BNT system. The enhanced *W_rec_* is attributed to the small grains and delays in polarization produced by La doping, whereas remnant polarization is decreased following Sr doping. Zhai et al. [96,97] utilized an A-site defect engineering method (nonstoichiometric ratio of Bi and Na) to reduce the electric conductivity and enhance the grain size, which resulted in a high *W_rec_* (5.63 J/cm^3^ and 3.72 J/cm^3^) and a high *η* (94% and 90.7%) in binary and ternary systems, such as 0.75Bi_0.58_Na_0.42_TiO_3_-0.25SrTiO_3_ and BNT-Bi_0.1_Sr_0.85_TiO_3_-KNbO_3_. Wu et al. [98] reported the incorporation of Sr_0.85_Bi_0.1□0.05_TiO_3_ (SBT) and NaNbO_3_ (NN) into a BNT system via a compositional design. The substitution of Sr^2+^ ions and A-site vacancies constructed RFEs on the basis of the order–disorder theory, enabling a high *W_rec_* of 3.08 J/cm^3^ and a high *η* of 81.4%. Liu et al. [99] presented an intrinsic defect and polarization mechanism in A-site-deficient 0.66(Bi_0.5_Na_0.5_)TiO_3_-0.06BaTiO_3_-0.28(Bi*_x_*Sr_1–3*x*/2_)TiO_3_ (BNT-BT-BST) relaxors, favoring polarization behavior, which resulted in a *W_rec_* of 1.61 J/cm^3^ and a *η* of 90.5%. Hwang et al. [100] demonstrated the electric energy storage density and energy efficiency of (1 − *x*)Bi_0.5_(Na_0.8_K_0.2_)_0.5_TiO_3_-*x*Bi_0.2_Sr_0.7_TiO_3_ (BNKT-BST; *x* = 0.15–0.50) RFEs via a domain engineering method. The substitution of BST composition into the BNKT system can disturb the long-range ferroelectric order, reducing the dielectric maximum temperature *T_m_*, which leads to the formation of dynamic PNRs (Figure 8a). Additionally, the *T_m_* was shifted to a higher temperature with increasing frequency, signifying RFE behavior in BNKT-BST ceramics, which is supported by the modified Curie Weiss law (Figure 8b). The relaxor properties contribute to a higher *P_max_* and a lower *P_r_*, enhancing the BDS with the incorporation of BST, and leading to a high *W_rec_* of 0.81 J/cm^3^ and high *η* of 86.95% at an electric field of 90 kV/cm for a *x* = 0.45 composition (Figure 8c,d). Ma et al. [101] utilized a morphotropic phase boundary (MPB) 0.76Bi_0.5_Na_0.5_TiO_3_-0.24SrTiO_3_ (BNT-ST) RFE with the incorporation of AFE AN to a lower *P_r_* and retained the same *P_max_* in order to achieve a *W_rec_* of 2.03 J/cm^3^. Furthermore, lead-free KNaNbO3-based RFEs have been explored to enhance their energy storage properties. Yang et al. [102] reported composition-driven grain size to a sub-micrometer scale (~100–200 nm) to enhance the breakdown strength of (K_0.5_Na_0.5_)NbO_3−_*x*SrTiO_3_ (KNN-ST) RFEs, and showed a high *W_rec_* of 4.03 J/cm^3^ at 400 kV/cm. Similarly, KNN has been modified with BiFeO_3_, Sr(Sc_0.5_Nb_0.5_)O_3_, and Bi(Mg_2/3_Nb_1/3_)O_3_ ceramics, and high *W_rec_* values of 2 J/cm^3^, 2.60 J/cm^3^, and 4.08 J/cm^3^ were achieved [34,65,103,104]. Xie et al. [105] reported an ultra-high *W_rec_* of 8.73 J/cm^3^ and a high *η* of 80.1% in 0.68 NaNbO_3_-0.32Bi_0.5_Li_0.5_TiO_3_ ceramics, achieved via exploiting the stable orthorhombic FE phase instead of the AFE orthorhombic phase (Figure 9a,b). In addition, they introduced the AFE relaxor concept to discuss the energy storage performance of 0.78NN-0.24BNT systems. They reported that the local AFE was transformed/reversed into the FE phase at an electric field of 400 kV/cm, inducing a large *P_max_* (50 µC/cm^2^) and a low *P_r_* of 5 µC/cm^2^, which together provided an enhanced ultra-high *W_rec_* of 12.2 J/cm^3^ and a high *η* of 69% at an electric field of 680 kV/cm, as shown in Figure 9c,d [65]. The energy storage properties of ceramic-based dielectric materials are listed in Table 1.

### 3.2. Ceramic Films

In Section 3.1.4, we presented lead-free RFE materials, which are good candidates for energy storage device applications, owing to their ultra-high energy storage density, excellent BDS, and eco-friendliness. However, the miniaturization of electronic devices is necessary for real-world applications, such as hybrid electric vehicles, defense artillery, and smart and wearable electronics [145,146,147]. Therefore, thin/thick film capacitors (e.g., RFEs) have received significant attention in developing high-performance ceramic capacitors for energy storage as compared to bulk ceramic capacitors (LDs, FEs, and AFEs) [1,148,149,150]. Interestingly, these film capacitors have a higher BDS due to less defects, which results in a high energy density. In addition, thin/thick film capacitors are promising for miniaturized electronic devices due to their uniform and highly dense microstructure. The thickness of ceramic capacitors plays an important role in determining the BDS. The thickness/volume ratio of a film capacitor determines its energy storage capacity. Moreover, ceramic capacitor devices with a higher BDS are safe for operation at high voltages and have a smaller likelihood of device failure [6,151].

RFE film-based dielectric capacitors that adopt various strategies for energy storage have been investigated [152,153,154,155,156,157,158,159,160,161,162,163,164,165,166,167,168,169]. Zhang et al. [170] improved the energy storage performance via a small amount of Mn doping (1 mol.%) in 0.70BNT-0.3ST RFE thin films. Mn^2+^ ions induce an intrinsic restoring force and enable the reversible domain switching and slim *P-E* loops (Δ*P*~56 µC/cm^2^), resulting in a high *W_rec_* of 27 J/cm^3^. The same amount of Mn in 0.6ST-0.4BNT thin films yielded a high *W_rec_* of 33.58 J/cm^3^ at a BDS of 3134 kV/cm, owing to reduced oxygen vacancies [171]. Interestingly, BNT-BT has shown excellent dielectric properties at the MPB between the coexistence of a rhombohedral FE phase and a tetragonal AFE phase for x = 0.06. Peng et al. [172] reported an ultra-high *W_rec_* of 154 J/cm^3^ via the co-doping of La and Zr in 0.94BNT-0.06BT RFE thin films. The La dopant plays a critical role in enhancing the relaxor properties, whereas the Zr dopant was utilized to control the transition temperature. Pan et al. [173] reported an energy density of 70 J/cm^3^ in 0.55BiFeO_3_–0.45SrTiO_3_ (BF-ST) films via a domain engineering method. The substitution of ST into BF can transform the micrometer-scale FE domains into highly dynamic PNRs, resulting in a high energy storage density in the BF-ST films. In addition, they demonstrated that the coexistence of rhombohedral and tetragonal nanodomain structures in a cubic paraelectric matrix creates a flattened domain-switching pathway in BF-BT-ST films, which minimizes hysteresis loss and delivers an energy density of 112 J/cm^3^ [152]. Pan and co-workers carried out phase-field simulations in order to choose the proper combination of BF and BT with Sm doping to achieve high energy storage. These simulations were helpful in designing super-paraelectric RFEs with unique and smaller size nanodomains in a Sm-doped BF-BT system, which generated an ultra-high *W_rec_* of 152 J/cm^3^ and a high *η* of 90% [174]. The energy storage properties of the ceramic films are summarized in Table 2.

### 3.3. Multilayer Ceramic Capacitors

MLCCs have received extensive attention in the field of energy storage capacitor applications due to their ultra-high energy density, efficiency, and fast charge–discharge rates [175,176,177,178,179]. In recent years, the energy storage performance was improved in RFE Bi_0.5_Na_0.5_TiO_3_ and AFE AgNbO_3_-based lead-free ceramics, attaining energy densities of 2.7 J/cm^3^ and 4.2 J/cm^3^, respectively [26,177,178,180,181,182,183,184,185,186]. However, high energy dissipation and poor stability are attributed to the AFE to FE phase transition, which are the main drawbacks of AFEs limiting their practical applications. In this regard, Li et al. [5] demonstrated 0.55(Bi_0.5_Na_0.5_)TiO_3_ (BNT)-0.45(Bi_0.2_Sr_0.7_)TiO_3_ (BST) MLCCs and improved their energy density and efficiency by combining RFE and AFE features. The RFE exhibits highly dynamic polar nano-regions and disrupts the long-range ferroelectric order, which results in a hysteresis-free *P-E* loop. The RFE BST displaying a diffused phase transition was utilized with BNT to obtain RFE features, and is expected to reduce polarization and the high Δ*P*.

**Figure 10:**
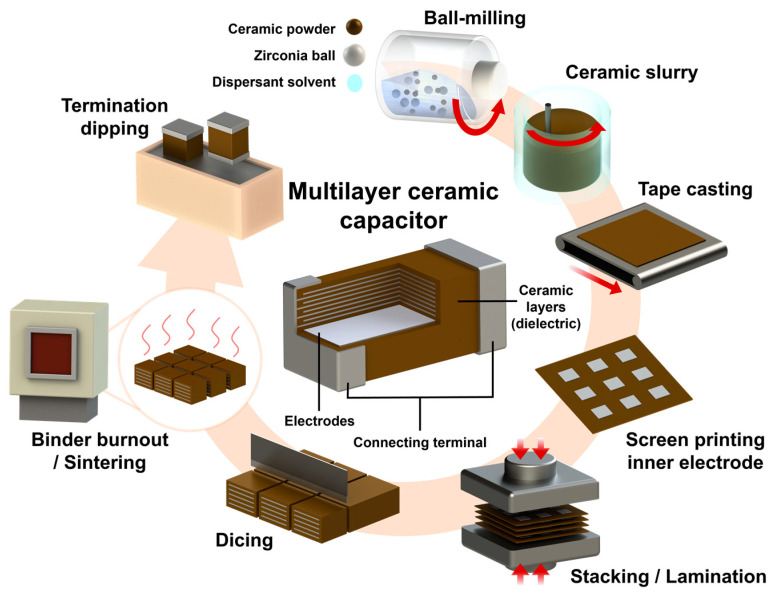
Schematic diagram of the MLCC fabrication process.

MLCCs have been fabricated using the tape-casting technique, which has two main advantages as follows: (i) The MLCC layers offer low porosity and a fine grain size, leading to a high *E_b_*. (ii) A higher E_b_ is expected in the MLCC compared to conventional ceramic capacitors because the *E_b_* increases with the decreasing layer thickness. The fabrication process of the MLCCs entails various stages, such as ball milling, slurry formation, tape casting, screen printing, stacking/lamination, dicing, sintering, and termination dipping. Figure 10 presents a schematic illustration of the MLCC fabrication process [187,188]. The ceramic powders were ball milled, slurry dried, and calcined. This calcined powder was re-milled with a dispersant (ethyl methyl ketone), binder (poly(propylene carbonate)), and plasticizer (butyl benzyl phthalate). Furthermore, a slurry was used to prepare thick films using a tape-casting process. The films were stacked layer by layer with inner printed Pt electrodes and then sintered at the desired temperatures to obtain the MLCCs. Lastly, the sintered samples were polished to terminate the opposite ends of the MLCC, and silver paste was coated to form the outer electrodes for electrical characterizations.

**Figure 11:**
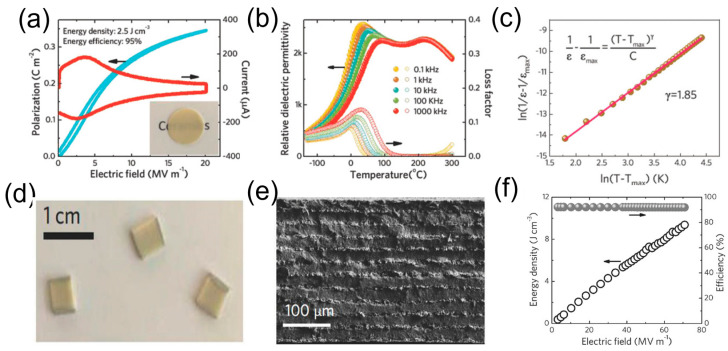
(**a**) Polarization (blue curve) and current (red curve) response are a function of the electric field (the inset shows a picture of the bulk sample), (**b**) the temperature variation of relative dielectric permittivity and loss factor, and (**c**) $ln\left(T-T_{max}\right)$ versus ln1ε−1εmax of 0.55(Bi_0.5_Na_0.5_)TiO_3_-0.45(Bi_0.2_Sr_0.7_)TiO_3_ ceramics. (**d**,**e**) A photograph and SEM image of MLCC of 0.55(Bi_0.5_Na_0.5_)TiO_3_-0.45(Bi_0.2_Sr_0.7_)TiO_3_. (**f**) Energy density and efficiency versus the applied electric field of 0.55(Bi_0.5_Na_0.5_)TiO_3_-0.45(Bi_0.2_Sr_0.7_)TiO_3_ MLCC. Reproduced with permission [5]. Copyright 2018, Wiley-VCH.

Figure 11a shows the unipolar polarization and current versus electric field curve for a 0.55(Bi_0.5_Na_0.5_)TiO_3_ (BNT)-0.45(Bi_0.2_Sr_0.7_)TiO_3_ (0.55BNT-0.45SBT) ceramic sample. It exhibited a high energy storage density of 2.5 J/cm^3^ and a high efficiency of 95% at a high breakdown field of 20 MV/m. The temperature dependence of the relative dielectric permittivity and the loss factor of the 0.55BNT-0.45SBT sample are shown in Figure 11b. The dielectric maximum temperature (*T_m_*) shifted towards higher temperatures, and the dielectric peaks diffused with increasing frequency, revealing the formation of high-dynamic polar nanoregions (PNRs); such materials are called relaxor ferroelectrics [189,190]. The degree of the diffuseness (*γ*) of the 0.55BNT-0.45SBT sample is found to be 1.85, indicating strong relaxor behavior (Figure 11c). Due to the formation of PNRs, the 0.55BNT-0.45SBT ceramic sample exhibits a high relative dielectric permittivity, high energy density, and high energy efficiency. To achieve ultrahigh energy density, 0.55BNT-0.45SBT MLCCs were fabricated using the tape-casting method. They consist of 10 dielectric layers with a total thickness of 200 µm and an inner electrode area of 6.25 mm^2^ (each layer has a thickness of 20 µm), as shown in Figure 11d. The surface morphology of the MLCC is shown in Figure 11e. The breakdown electric field was increased to 72 MV/m due to the advantages of the MLCCs fabricated using the tape-casting method, which offers low porosity and a fine grain size when compared to their counterpart bulk ceramics [191]. In general, the breakdown strength of the ceramics increases as the layer thickness decreases, as observed in many ceramics [192,193]. Figure 11f shows the energy density and efficiency as a function of the electric field for 0.55NBT-0.45SBT MLCCs. The energy storage density of these MLCCs exhibited a high *W_rec_* of 9.5 J/cm^3^ and a *η* of 92% at 72 MV/m. These results indicate that combining the antiferroelectric and relaxor properties of MLCCs is a promising approach for improving the energy storage responses in order to meet the requirements of advanced energy storage devices. In recent years, various strategies, including controlled phase [177], chemical homogeneity [178], grain orientation [194], combining antiferroelectric and relaxor properties [5], heterovalent doping [195], and two-step sintering [196], etc., were adopted to enhance the energy storage performance of MLCCs, as summarized in Table 3. In general, as the layer thickness decreases, the BDS of solid dielectrics increases [197]. Thin films show a higher BDS when compared to bulk ceramics and MLCs due to the minimal thickness and less defects, but they have limitations in energy storage density and efficiency. MLCCs have a lower BDS than thin films, but they have other advantages, such as a compact size, a balance between the BDS and energy storage, and good temperature stability, which play an important role in practical applications, especially in pulsed power systems.

## 4. Challenges and Future Prospects

With the discovery of new materials and strategies, the energy storage density of bulk ceramics, thin films, and MLCCs has been greatly improved to 12, 159, and 52 J/cm^3^, respectively, as summarized in Table 1, Table 2 and Table 3. Even with the tremendous advancements, there are still certain challenges in real-world applications. Dielectric ceramics with a high energy storage density of more than 8 J/cm^3^ with a high efficiency of over 90% are still scarce and cannot meet the demands of miniature advanced electronic and electric power systems. To achieve a high energy storage density in dielectrics, researchers mostly focused on the enhancement of Δ*P* and *E_b_*. Extensively utilized strategies for enhancing *E_b_* are reducing the grain size with homogeneous microstructures, stimulating electrical homogeneity, raising resistance, enhancing thermal conductivity, and lowering dielectric losses. These strategies can be implemented by employing advanced sintering procedures, adding sintering aids, employing two-step sintering, adjusting the heating/cooling rate/holding time, and making composite materials. However, effective strategies for further improving the *E_b_* remain limited. To obtain a high Δ*P*, the most popular method is to choose a host material with strong ferroelectricity and then decrease its *P_r_* via composition doping. On the other hand, select a host material with a modest *P_r_* and then add a secondary compound to enhance its *P_max_*. However, it remains challenging to achieve both a high *P_max_* and a low *P_r_* in these solid solutions. The domain engineering method allows for the fabrication of dielectrics with a low *P_r_* and a moderate Δ*P* via producing PNRs/nanodomains. However, the *P_max_* value remains low, restricting the raise of the *W_rec_*. Recently introduced local region design techniques, such as designing local regions with polarization-field response behavior or constructing local regions with polymorphic PNRs via phase structure regulation, will be an excellent choice for developing dielectrics with a high *P_max_* and a low *P_r_*.

Developing dielectric materials with a high *W_rec_* and *η* remains the path of future research. In addition, the trade-off between the *W_rec_* and *η* and the contradiction between the *ε_r_* and the *E_b_* must be resolved. New materials, new manufacturing techniques, and new design strategies must be discovered in order to achieve these goals. Further research is needed to understand the underlying mechanisms, such as sample sintering processes, dielectric breakdown strength, and dielectric polarization responses in local regions, ultimately developing a profound understanding of the material–structure–property relationship of dielectric materials for energy storage. In addition to developing a single material, more attention should be paid to composite materials, for instance, ceramic/ceramic composites, ceramic/glass composites, ceramic/polymer composites, and ceramic/glass/polymer composites, because it is challenging to develop a single material with a high *P_max_*, a low *P_r_*, a high *E_b_*, low dielectric loss, and excellent thermal stability/fatigue. Dielectric capacitors with an easy preparation technique, a simple chemical composition, and a low sintering temperature are still in great demand for practical applications. To fabricate new materials, advanced synthesis techniques (two-step sintering and pressure-assisted sintering), comprehensive characterizations (aberration-corrected scanning transmission electron microscope and piezoelectric force microscopy), various control strategies (nanodomain and grain size engineering), and theoretical calculations (machine learning and phase-field simulations) should be employed.

Ceramic-based films show an enormous performance when compared to bulk ceramics in terms of the energy storage density and dielectric breakdown strength. The energy storage properties of ceramic films have been enhanced via various methods, including solid solution formation, layered films with particular configurations (such as sandwich structures, positive/negative gradient compositions), the interface design of films/electrodes, the lattice/strain engineering of films/substrates, and more. Among them, similar to bulk ceramics, the fundamental solution is to deeply understand the inherent nature of whether AFEs/RFEs. Developing films for energy storage is challenging due to their restricted thickness and low absolute energy content. Developing various stratification and flexible scroll technologies is a viable solution for increasing the volume without losing their characteristics. Technological simplicity has the ability to accelerate manufacturing processes and boost automation, thus leading to cost savings and innovation.

MLCCs play an important role in dielectric energy storage. The macroproperties of MLCCs are mostly determined by the thickness of the dielectric layer in addition to their composition. Developing layer thinning techniques is crucial for increasing the energy density per volume. Furthermore, the expensive cost of metal electrodes, such as Au, Pt, and Ag, hinders the commercialization of MLCCs. Low-cost electrodes must be compatible with dielectrics, taking into account the sintering temperature, metal melting temperature, and interface reaction. Therefore, economical electrodes and appropriate cofire techniques should be developed. Since different metals are typically doped to internal and terminal electrodes in most cases, the method for joint connections between these electrodes should be a crucial consideration.

## 5. Conclusions

Dielectric materials with high power density and ultra-fast discharge rates are becoming increasingly significant in advanced electronic devices and pulsed power systems. Currently, dielectric energy-storage materials are limited in their applications due to their low energy density. Therefore, dielectric materials with excellent energy storage performance are needed. In this review paper, we discuss the fundamental concepts for energy storage in dielectric capacitors, including principles, key parameters, and influence factors for enhancing the energy storage properties. In addition, we summarize the recent progress of dielectrics, such as bulk ceramics/composites, ceramic films, and multilayer ceramic capacitors, followed by the best strategies, such as chemical modification, grain refinement, and defect engineering, for achieving a higher energy density/BDS and higher energy efficiency in dielectric materials for applications in pulsed power systems. Moreover, we present challenges and opportunities for future energy storage dielectric materials.

## Figures and Tables

**Table 1:** Energy storage properties of ceramic-based dielectric bulk materials.

Bulk Ceramics	Composition	*W_rec_* (J/cm^3^)	*η* (%)	*E_b_* (kV/cm)	Refs.
	Ca_0.5_Sr_0.5_(Ta_0.024_Ti_0.97_)O_3_-2 wt%SiO_2_	2	96	360	[106]
LDs	Ca_0.5_Sr_0.5_Ti_0.97_Sn_0.03_O_3_	2.06	95	330	[107]
	Ca_0.5_Sr_0.5_Ti_0.9_Zr_0.1_O_3_	2.05	85	390	[108]
	(Ca_0.5_Sr_0.5_)_0.8875_La_0.075_TiO_3_	2.07	93	370	[109]
	0.7(Bi_0.5_K_0.5_TiO_3_)-0.3SrTiO_3_	2.31	77.7	190	[110]
	(Ca_0.5_Sr_0.5_)_0.99_Mg_0.01_TiO_3_	2.88	90	460	[111]
	Ca_0.5_Sr_0.5_Ti_0.85_Zr_0.15_O_3_	3.37	96	440	[112]
	0.9(Sr_0.7_Bi_0.2_TiO_3_)-0.1Bi(Ni_2/3_Nb_1/3_)O_3_	3.71	97	340	[113]
	Ca-doped SrTiO_3_	1.95	72.3	333	[41]
	Zr doped Sr_0.98_Ca_0.02_TiO_3_	2.77	77.7	-	[47]
	Mg-doped SrTiO_3_	1.86	72.3	362	[45]
	CaZrO_3_-0.05SrTiO_3_	5	-	1000	[48]
	0.8CaTiO_3_-0.2CaHfO_3_	9	-	1200	[43]
FEs	Ba_0.3_Sr_0.7_TiO_3_	0.23	95.7	90	[49]
	Nd and Mn-doped Ba_0.7_Sr_0.3_TiO_3_	0.41	84.6	110.6	[50]
	1.6 wt% ZnO doped Ba_0.3_Sr_0.7_TiO_3_	3.9	-	40	[33]
	(BaCa)(ZrTi)O_3_	1.28	-	243	[52]
	BiFeO_3_–BaTiO_3_–Bi(Mg_2/3_Nb_1/3_)O_3_	1.27	-	110	[114]
	BaTiO_3_–Bi(Zn_2/3_(Nb_0.85_Ta_0.15_)_1/3_)O_3_	2.06	78	180	[115]
	0.9BaTiO_3_-0.1Bi(Mg_1/2_Hf_1/2_)O_3_	3.38	87	240	[116]
AFEs	(Pb_0.91_Ba_0.045_La_0.03_)(Zr_0.6_Sn_0.4_)O_3_	8.16	92.1	340	[117]
	0.84(Bi_0.5_Na_0.5_)TiO_3_-0.16KNbO_3_	5.2	88	310	[118]
	Ag_0.76_La_0.08_NbO_3_	7.01	77	476	[119]
	Ag_0.97_Nd_0.01_Ta_0.20_Nb_0.80_O_3_	6.5	71	370	[120]
	NaNbO_3_-Bi(Zn_2/3_Nb_1/3_)O_3_	2.4	90	300	[121]
	0.85(NaNbO_3_)-0.15(Bi(Ni_2/3_Nb_1/3_)O_3_)	3.31	80.9	440	[122]
	(Na_0.41_La_0.09_)(Nb_0.82_Ti_0.18_)O_3_	6.5	66	550	[123]
	0.75[0.90NaNbO_3_-0.10Bi(Mg_0.5_Ta_0.5_)O_3_]0.25(Bi_0.5_Na_0.5_)_0.7_Sr_0.3_TiO_3_	8	90.4	800	[124]
	0.68NaNbO_3_-0.32(Bi_0.5_Li_0.5_)TiO_3_	8.73	80.1	-	[105]
	0.76NaNbO_3_-0.24(Bi_0.5_Na_0.5_)TiO_3_	12.2	69	680	[65]
RFEs	0.93BaTiO_3_-0.07YNbO_4_	0.61	87	173	[125]
	0.65Bi1_.05_FeO_3_-0.35BaTiO_3_-(BiNa_0.84_K_0.16_)_0.48_Sr_0.04_TiO_3_	0.81	60	100	[126]
	0.93BaTiO_3_-0.07Sr(Zn_1/3_Nb_2/3_)O_3_	1.45	83.12	260	[127]
	0.88BaTiO_3_-0.12Bi(Ni_2/3_Nb_1/3_)O_3_	2.09	95.9	220	[128]
	0.02Ce-doped 0.65BaTiO_3_-0.35Sr_0.7_Bi_0.2_TiO_3_	2.57	81.3	330	[129]
	(Ba_0.65_Sr_0.24_5Bi_0.07_)_0.99_Nd_0.01_TiO_3_	4.2	80	460	[130]
	0.85(0.95Bi_0.5_Na_0.5_TiO_3_-0.05SrZrO_3_)-0.15NaNbO_3_	3.14	79	230	[131]
	Na_0.25_Bi_0.2_5Sr_0.5_)(Ti_0.8_Sn_0.2_)O_3_	3.4	90	310	[132]
	0.88Bi_0.47_Na_0.47_Ba_0.06_TiO_3_-0.12CaHfO_3_	4.2	66.7	280	[133]
	0.75(Bi_0.45_La_0.05_Na_0.5_)_0.94_Ba_0.06_TiO_3_-0.25Sr_0.8_Bi_0.1_□_0.1_Ti_0.8_Zr_0.2_O_2.95_	3.84	90.8	330	[134]
	0.5(Na_0.5_Bi_0.5_TiO_3_)-0.5(Sr_0.85_Sm_0.1_TiO_3_)	5.02	90	422	[135]
	0.8Bi_0.5_Na_0.5_TiO_3_-0.2SrNb_0.5_Al_0.5_O_3_	6.64	96.5	520	[136]
	0.70Bi_0.5_Na_0.5_TiO_3_-0.30SrNb_0.5_Al_0.5_O_3_	6.78	89.7	572	[137]
	0.85K_0.5_Na_0.5_NbO_3_-0.15Bi(Li_0.5_Ta_0.5_)O_3_	1.1	56	151	[138]
	0.91K_0.5_Na_0.5_NbO_3_-0.09SrZrO_3_	2.81	80	370	[139]
	0.9(K_0.5_Na_0.5_)NbO_3_-0.1Bi(Zn_2/3_Nb_1/3_)O_3_	4.01	97.1	326	[140]
	[(Na_0.5_K_0.5_)_0.91_Li_0.03_](Nb_0.88_Sb_0.06_)O_3_-0.06Bi(Zn_1/2_Zr_1/2_)O_3_	4.85	88.2	480	[141]
	0.85K_0.5_Na_0.5_NbO_3_-0.15Bi(Zn_2/3_Ta_1/3_)O_3_	6.7	92	600	[142]
	0.90K_0.5_Na_0.5_NbO_3_-0.10Bi(Zn_2/3_(Nb_0.85_Ta_0.15_)_1/3_)O_3_	7.4	78	800	[143]
	0.85K_0.5_Na_0.5_NbO_3_-0.15Bi(Ni_0.5_Zr_0.5_)O_3_	8.09	88.46	870	[144]

**Table 2:** Energy storage properties of ceramic films.

Film Composition	*W_rec_* (J/cm^3^)	*η* (%)	*E_b_* (kV/cm)	Refs.
BiFeO_3_-BaTiO_3_-SrTiO_3_	112	80	5.3 × 10^3^	[152]
0.5Ba(Zr_0.2_Ti_0.8_)O_3_–0.5(Ba_0.7_Ca_0.3_)TiO_3_ (BCZT)	99.8	71	750	[153]
0.6(Bi_0.5_Na_0.5_)TiO_3_–0.4Bi(Ni_0.5_Zr_0.5_)O_3_	50.1	63.9	2200	[154]
Mn-doped 0.97(0.93Na_0.5_Bi _0.5_TiO_3_-0.07BaTiO_3_)-0.03BiFeO_3_	81.9	64.4	2285	[155]
Mn-doped 0.55(0.94Na_0.5_Bi_0.5_TiO_3_-0.06BaTiO_3_)-0.45SrTiO_3_	76.1	80	2813	[156]
Ba(Zr_0.35_Ti_0.65_)O_3_	65.1	72.9	6.15 × 10^3^	[157]
Sn-doped In_2_O_3_/BaZr_0.35_Ti_0.65_O_3_	40.6	68.9	4.23 × 10^3^	[158]
0.9Bi_0.2_Sr_0.7_TiO_3_–0.1BiFeO_3_	48.5	47.57	4800	[159]
Mn-doped BiFeO_3_–BaTiO_3_	80	78	3.1 × 10^3^	[160]
0.5(Bi_0.5_Na_0.5_)TiO_3_-0.5Bi(Zn_0.5_Zr_0.5_)O_3_	40.8	64.1	1500	[161]
0.88Ba_0.55_Sr_0.45_TiO_3_–0.12BiMg_2/3_Nb_1/3_O_3_	86	73	5 × 10^3^	[162]
0.3Bi(Fe_0.95_Mn_0.05_)O_3_-0.7(Sr_0.7_Bi_0.2_)TiO_3_	61	75	3000	[163]
(Na_0.8_K_0.2_)_0.5_Bi_0.5_TiO_3_/0.6(Na_0.8_K_0.2_)_0.5_Bi_0.5_TiO_3_-0.4SrTiO_3_	73.7	68.1	2308	[164]
Na_0.5_Bi_3.25_La_1.25_Ti_4_O_15_/BaBi_3.4_Pr_0.6_Ti_4_O_15_	159.7	70	3450	[165]
Mn-doped 0.65(0.94Na_0.5_Bi_0.5_TiO_3_–0.06BaTiO_3_)–0.35SrTiO_3_	56	66	2738	[166]
Sr_0.975_(Bi_0.5_Li_0.5_)_0.025_Ti_0.99_Mn_0.01_O_3_	47.7	66.5	3307	[167]
Ba(Zr_0.1_Ti_0.9_)O_3_	15.5	69.8	1500	[168]
HfO_2_/Al_2_O_3_/ZrO_2_	54.3	51.3	5000	[169]

**Table 3:** Energy storage properties of MLCCs.

MLCC Composition	Thickness/No. of Active Layers	*W_rec_* (J/cm^3^)	*η* (%)	*E_b_* (kV/cm)	Refs.
0.75(Bi_1-x_Nd_x_)FeO_3_-0.25BaTiO_3_ (x = 15%)	32 µm/9	6.74	77	540	[177]
0.87BaTiO_3_-0.13Bi(Zn_2/3_(Nb_0.85_Ta_0.15_)_1/3_)O_3_	17 µm/10	8.13	95	750	[196]
0.55(Bi_0.5_Na_0.5_)TiO_3_-0.45(Bi_0.2_Sr_0.7_)TiO_3_	20 µm/10	9.5	92	720	[5]
0.62BF–0.3BT-0.08NdZn_0.5_Zr_0.5_O_3_	16 µm/7	10.5	87	700	[178]
Sm_0.05_Ag_0.85_Nb_0.7_Ta_0.3_O_3_	10 µm	14	85	1450	[195]
0.5BiFeO_3_-0.4SrTiO_3_-0.03Nb-0.1 BiMg_2/3_Nb_1/3_O_3_	8 µm	15.8	75.2	1000	[198]
<111>Na_0.5_Bi_0.5_TiO_3_-Sr_0.7_Bi_0.2_TiO_3_	20 µm/10	21.5	65	103 × 10^3^	[194]
Ba_0.3_Sr_0.7_TiO_3_/0.85BaTiO_3_-0.15Bi(Mg_0.5_Zr_0.5_)O_3_	230 nm/8	30.64	70.93	3000	[199]
Ba_0.7_Ca_0.3_TiO_3_-BaZr_0.2_Ti_0.8_O_3_	100 nm/8	52.4	72.3	4.5 × 10^3^	[176]

## Data Availability

Not applicable.
